# Type of Evidence Behind Point-of-Care Clinical Information Products: A Bibliometric Analysis

**DOI:** 10.2196/jmir.1539

**Published:** 2011-02-18

**Authors:** Andrea M Ketchum, Ahlam A Saleh, Kwonho Jeong

**Affiliations:** ^2^Department of BiostatisticsGraduate School of Public HealthUniversity of PittsburghPittsburgh, PAUnited States; ^1^Health Sciences Library SystemUniversity of PittsburghPittsburgh, PAUnited States

**Keywords:** Databases, Factual, Bibliometrics, Medical Informatics, Evidence-based Medicine

## Abstract

**Background:**

Point-of-care (POC) products are widely used as information reference tools in the clinical setting. Although usability, scope of coverage, ability to answer clinical questions, and impact on health outcomes have been studied, no comparative analysis of the characteristics of the references, the evidence for the content, in POC products is available.

**Objective:**

The objective of this study was to compare the type of evidence behind five POC clinical information products.

**Methods:**

This study is a comparative bibliometric analysis of references cited in monographs in POC products. Five commonly used products served as subjects for the study: ACP PIER, Clinical Evidence, DynaMed, FirstCONSULT, and UpToDate. The four clinical topics examined to identify content in the products were asthma, hypertension, hyperlipidemia, and carbon monoxide poisoning. Four indicators were measured: distribution of citations, type of evidence, product currency, and citation overlap. The type of evidence was determined based primarily on the publication type found in the MEDLINE bibliographic record, as well as the Medical Subject Headings (MeSH), both assigned by the US National Library of Medicine. MeSH is the controlled vocabulary used for indexing articles in MEDLINE/PubMed.

**Results:**

FirstCONSULT had the greatest proportion of references with higher levels of evidence publication types such as systematic review and randomized controlled trial (137/153, 89.5%), although it contained the lowest total number of references (153/2330, 6.6%). DynaMed had the largest total number of references (1131/2330, 48.5%) and the largest proportion of current (2007-2009) references (170/1131, 15%). The distribution of references cited for each topic varied between products. For example, asthma had the most references listed in DynaMed, Clinical Evidence, and FirstCONSULT, while hypertension had the most references in UpToDate and ACP PIER. An unexpected finding was that the rate of citation overlap was less than 1% for each topic across all five products.

**Conclusions:**

Differences between POC products are revealed by examining the references cited in the monographs themselves. Citation analysis extended to include key content indicators can be used to compare the evidence levels of the literature supporting the content found in POC products.

## Introduction 

Studies of the information-seeking practices of physicians suggest that the use of electronic resources for clinical care has increasingly become a standard method of information access alongside traditional methods of textbook and colleague contact [1-4]. Ease of access plays an important role in the popularity of electronic resources for answering clinical questions at the bedside, but lack of time is one of the main factors found to contribute to limited use of evidence-based medicine (EBM) by residents and consulting physicians [2,4-7]. Electronic information resources improve timely access in the form of desktop or mobile product applications by presenting information to clinicians in a summarized format.

Point-of-care (POC) products, the subject of this study, are resources we define as electronic bedside information tools that provide summarized medical information for use by health care professionals. A few examples of these products are UpToDate, eMedicine, DynaMed, and Clinical Evidence. Other arbitrary designations used to refer to these products are e-textbooks, evidence-based resources, and decision support tools. Haynes’ 6S pyramid model of preappraised resources, a modification of the 5S model, describes these products as summaries [8,9]. Many of these resources do claim to be evidence based in their descriptions or policy statements. However, the foundation for the evidence basis is not always transparent. 

To date, this class of information products has been studied in terms of features, usability, impact on health outcomes, scope of coverage, and ability to answer clinical questions. POC products have been ranked by user “perception of content” [10], “perceived usefulness” [11], and satisfaction with interface and overall search experience.

The ability of electronic information resources to help clinicians find correct answers at the point of care at the time of need is critical. Research has expanded beyond documenting variation in user experience and satisfaction to examining the impact POC products have on clinical practice and patient outcomes.

Seeking to measure the impact of electronic information products on patient outcomes, Bonis and colleagues compared acute care hospitals with and without access to UpToDate and found that hospitals with access to UpToDate were associated with better patient care quality and outcomes performance, and shorter lengths of stay [12].

In an observational study of residents and specialists comparing UpToDate versus PubMed, Hoogendam and colleagues concluded that UpToDate was the preferred source and answered more patient-related questions, but included the many complexities of the findings in the discussion [13]. Other observational studies have shown that third-year family medicine residents at a 5-hospital residency program directed only 3% (15/532) of their clinical questions to electronic resources [14], while emergency department physicians received 29%(36/126) of their answers from electronic resources [15].

Alper et al showed that the percentage of questions answered by 14 individual electronic information resources may range widely but, in combination, much higher rates could be achieved [16]. Following up on that work, Fenton and Badgett examined the scope of coverage and overlap between two information resources, UpToDate and US National Guideline Clearinghouse [17].

In a 2004 comparison study, three EBM resources were used to answer sets of complex and general clinical questions. A combined total of 35% (28/80) of the test questions were left unanswered [18]. In another study published in 2005, clinicians using six electronic information sources increased the number of correctly answered questions by 21%, and were also more likely to correct their wrong answers [19]. Alper et al found that primary care physicians using DynaMed answered more clinical questions (263/347, 75.8%) and found more answers that changed clinical decisions (224/347, 64.6%) than did the comparison group’s 15 typical information resources (209/351, 59.5%), without increasing search time [20].

McKibbon and Fridsma examined the effectiveness of electronic information resources chosen by 23 primary care physicians in the United States and Canada [21]. This study found that, when physicians used their own information resources, they correctly answered only 42% of their questions versus 39% before searching. In some cases, participants changed correct answers to incorrect. McKibbon and Fridsma [21] concluded that

...the evidence base of the resources must be strong and current...We need to evaluate them well to determine how best to harness the resources to support good clinical decision making.

To our knowledge, very few studies focus on evaluation of the content that supports disease POC products. For example, Trumble and colleague’s 2006 product evaluation measured features and usability, but also added weighted factors for specific evidence features for the purpose of ranking the products: graded evidence, summary of evidence, updating, authorship, references, and within-text bibliography at the end [22]. The weighted evidence feature resulted in a ranked list of POC products. Farrell evaluated the five most-used resources as identified by a survey of 52 Canadian health librarians [23]. Usability and comprehensiveness of each product were tested, with level of evidence noted if included in the answers retrieved. Banzi et al recently reviewed 18 products in depth using a scoring instrument and found differences in the volume of coverage, EBM content, and editorial methodology [24]. A 2009 evaluation by Abernethy et al, which examined the reliability of compendia methods for off-label oncology indications, found discordance and lack of currency in cited references [25].

Our objective was to use a bibliometric approach using citation and content indicators as another method to evaluate a set of POC resources. 

“Bibliometrics is the quantitative study of physical published units, or of bibliographic units, or of the surrogates for either,” as defined by Broadus in a 1987 paper outlining the history of attempts to describe the new term [26]. Citation analysis falls within that broader term, and “...involves the construction and application of a series of indicators of the ‘impact’, ‘influence’ or ‘quality’ of scholarly work, derived from citation data, i.e. data on references cited in footnotes or bibliographies of scholarly research publications” [27]. 

Citation analysis for evaluation of groups and individuals and to describe broad scientific developments has been scrutinized since the introduction of *Science Citation Index* in 1961 [28,29]. Implications of the citation behavior of authors have been studied and defined, with much concern regarding its administrative application to individual scientists [30-32].

The indicator associated with citation analysis is the impact factor, which Garfield [33] describes as a measure of utility:

They provide an objective measure of the utility of impact of the scientific work. They say nothing about the nature of the work, nothing about the reason for its utility or impact. Those factors can be dealt with only by content analysis of the cited material and the exercise of knowledgeable peer judgment.

This study expands citation analysis beyond count indicators, such as simply counting the reference to certain articles or authors in the monographs and/or the overlap analysis, to include pertinent content indicators from the bibliographic or citation record, specifically the MEDLINE publication type and publication year. Our intention is that the addition of the publication type indicator, in Garfield’s words, suggests “something about the reason for its utility or impact” [33]. The aggregate of the indicators and the impact on the quality of the POC product are also of interest in this investigation, rather than the individual authors or articles.

## Methods 

We measured four indicators in this study: distribution of citations, type of evidence, product currency, and citation overlap.

Distribution of citations is the number of citations within each POC product and as distributed across the disease topics. It was measured to give a sense of the depth of coverage within each product, as well as across all products. This measure was also used to compare topic coverage within products and across the five different products.

When an evidence-based recommendation for treatment or other aspect of care is made, an original source should be cited to support the recommendation. Our proxy for evidence was publication type. We chose this surrogate as it can be readily compared and evaluated in terms of the types of evidence typically found in evidence hierarchies through pyramid representations or grading schemes. This approach was also more realistic and feasible given the time restrictions of the study**.**

Citation publication date is important because we want to know that these products are providing the most current information to users. Years were grouped in an every-3-years format with the exception of pre-2001 citations. When clinicians are searching for evidence relevant to current practice, they are less likely request retrieval of articles more than 10 years old. We therefore grouped pre-2001 into one category.

We also looked at citation overlap, as we expected to find significant overlap among products for the same topics. Citation overlap across products would give us an indication of consistency of content across products. 

### Topic Selection

(See Multimedia Appendix 1.)

Four final disease topics were selected for the study from the National Library of Medicine’s (NLM) Clinical Question Collection [34] through a three-step selection process. The topics were to be restricted to a small number in order to effectively manage the data display in this initial study. The first step in identifying questions from the collection was to randomly select numbers. A random integer generator produced 35 random numbers between 1 and 4654 [35]. The main topics from the corresponding 35 clinical questions from the NLM’s Clinical Question Collection were then examined for inclusion/exclusion criteria. Topics were required to be a main entry in each POC product, and drug/substance topics were excluded, as it is not uncommon for drug information content to originate from third-party licensed resources. We did not follow a rigid protocol to identify a topic as a main entry; reviewers simply checked to see that there was an article for each condition in the product. This resulted in 15 topics from the original 35. 

To further limit the number, we then compared the resultant 15 topics against the US Centers for Disease Control and Prevention’s top 10 leading causes of death for all people as listed in the 2007 Chartbook [36].The topic must be listed to be included in the study. There were 8 topics remaining after the second-phase screening. In the third phase of selecting topics, we excluded four additional topics (tuberculosis, influenza, pneumonia, and follicular thyroid carcinoma) because they did not consistently cover the same scope across all five POC products. For example, the topic tuberculosis was represented by a single monograph in some products, while in others, diagnosis and other aspects were written as separate monographs.

### Product Selection

Two authors (AK, AS) selected the products.

The products were selected from the top 10 rankings for evidence products in the Texas Consortiums Study in conjunction with the 5 selected resources in Farrel’s evaluation of POC resources [22, 23]. The POC resources common to both rankings were selected. These were ACP PIER, BMJ’s Clinical Evidence, UpToDate, and FirstCONSULT. An additional two resources from the Texas Consortium Study were selected that are similar to the other products in terms of function and presentation of materials: DynaMed and Essentials Evidence Plus (formerly known as InfoPOEMS), for a total of 6 POC products.

Due to anticipation of space limitation in reporting of the results data, we wanted to limit the test to a maximum of 5 products and therefore eliminated Essential Evidence Plus. Access was available through institutional subscriptions to all products except DynaMed, for which we obtained trial access.

### Data Collection

Data were collected during a 6-week period from mid-December 2008 to the end of January 2009. All four topic monographs were located in each of the POC resources. References accompanying a monograph entry were retrieved and saved in a Word document. Any additional reference lists such as “Further Reading” were not included.

All references were subsequently transferred into a reference management product. Along with typical elements of a citation, Medical Subject Heading (MeSH) indexing, the MEDLINE publication type assignment, and the PMID (a unique citation ID assigned to citations in PubMed) were collected. Citations with no PMID were assigned a unique identifier using a structured guide that we created. We attempted to verify Web citations and other nonjournal citations to obtain complete details as to the source and type of publication. For the purpose of consistency in this analysis, from this point forward all references and citations will be referred to as “citations.”

### Data Analysis

Topics were divided between two authors (AK, AS) for individual review of the citations. We discussed any uncertainty in review of the citations to come to consensus. No interrater reliability was calculated. To identify publication types for the citations we used the NLM’s MEDLINE indexing as a reference point in the citation classification process. (Note that when citations are input into MEDLINE, they are assigned index terminology including publication type from a standardized list of index terms.) We developed a protocol for assigning publication type based on this premise in conjunction with the Publication Type Classification System, which we devised to account for the limitations of simply using the MEDLINE publication type indexing (see Table 1). Further details on the protocol for assigning publication type may also be found in Multimedia Appendix 2. The development of the classification system scheme was guided by the hierarchy of evidence. While there is no standardized EBM pyramid hierarchy, we used the Dartmouth College Library’s EBM pyramid scheme [37] as a reference point to develop our Publication Type Classification System, as it is the pyramid used by many health sciences libraries when presenting evidence-based resources (see Table 1). Guideline developers may conduct systematic reviews as part of the synthesis of the guideline; however, they are not one and the same. Furthermore, not all guidelines are based on evidence (some are consensus opinion) and therefore we classified guidelines as a distinct publication type from systematic reviews.

**Table 1:** Publication type classification system

Publication type category	Details
Guideline	Includes both evidence-based and consensus guidelines
Systematic review	Included both systematic reviews and meta-analyses
Review	Narrative reviews, synopses, and other review types not considered systematic reviews
Primary research, other	Includes case-control, cross-sectional, cohort, case-series, unclear study type, or a combination study design
Randomized controlled trial	
Report	Used for government publications, statistical data reports, technology assessments, *Morbidity and Mortality Weekly Report* (MMWR), and working group/task force stand-alone reports
Animal study	Animal-only study
Other	Includes items such as letters, comments, editorials, abstracts, and books. Letters, comments, and editorials were examined in full text as needed to identify any reports of study data
Unknown	Unable to verify citation

Because systematic review is not a publication type in MEDLINE indexing, we further examined citations designated with the NLM publication type review to identify and classify into the systematic review category as appropriate. A set of criteria was devised for assigning citations to the systematic review category. The criteria were based on definitions of systematic reviews in JAMAevidence *Users’Guide to the Medical Literature* [38] and Cook’s 1997 article, “Systematic reviews: synthesis of best evidence for clinical decisions” [39]. The following is Cook’s definition [39]:

Systematic reviews are scientific investigations in themselves, with preplanned methods and an assembly of original studies as their“subjects.”They synthesize the results of multiple primary investigations by using strategies that limit bias and random error. These strategies include a comprehensive search of all potentially relevant articles and the use of explicit, reproducible criteria in the selection of articles for review. Primary research designs and study characteristics are appraised, data are synthesized, and results are interpreted.

Additional details regarding classification of systematic reviews are available in Multimedia Appendix 3.

Furthermore, we reviewed the abstracts and full text as needed for citations that were indexed with the publication types comments, letters, and/or editorial to determine whether any study data were reported. This decision was made since we encountered several instances where study data was reported in these publication types. These general MEDLINE publication types (comments, letters, and editorials) were then more descriptively reassigned to one of the publication type categories in Table 1. We re-examined all citations originally categorized as such and determined whether study data were reported based on meeting the following criteria that the authors created: sample/or subjects being studied were described characteristically/or in quantity (must have this); and traditional outlining of an abstract was present in the MEDLINE abstract or full text (for example, Objectives/Introduction/Problem; Methods/Subjects; Results; Discussion/Conclusion).

Data were analyzed using STATA version 10 (StataCorp LP, College Station, TX, USA).

## Results

Five POC products met inclusion criteria: UpToDate, FirstCONSULT, ACP PIER, Clinical Evidence, and DynaMed. The four final topics were hypertension, asthma, Carbon monoxide poisoning (CO poisoning), and hyperlipidemia. The last updated date for topics and products is noted in Table 2.

**Table 2:** Last updated date (day/month/year) for topics within point-of-care products

	ACP PIER	DynaMed	FirstCONSULT	UpToDate	Clinical Evidence
Carbon monoxide poisoning	1/30/2006	11/21/2008	9/28/2007	1/10/2008	1/23/08
Hypertension	11/26/2008	1/26/2009	8/24/2007	10/8/2008	2/1/2007
Asthma	11/25/2008	1/15/2009	8/23/2007	9/26/2008	Not available from authors
Hyperlipidemia	11/26/2008	1/13/2009	8/24/2007	5/27/2008	2/6/2008

**Figure 1:**
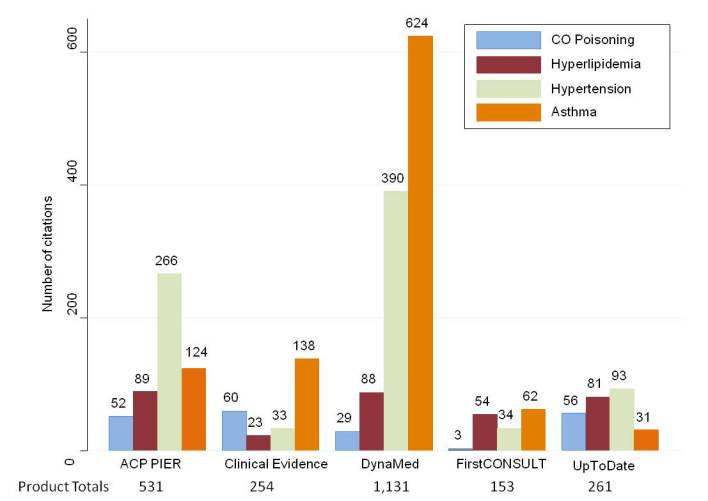
Number of citations for all topics within each point-of-care product

We retrieved a total of 2330 citations from the five POC products combined. As seen in Figure 1, almost half (1131/2330, 48.5%) of these citations originated from DynaMed, while only 6.6% (153/2330) of the citations from the total were obtained from FirstCONSULT. Figure 1 also illustrates the variation in the number of citations within each POC product across the four topics. For example, Clinical Evidence and DynaMed show greater proportions of citations for the topic asthma within each product, while ACP PIER and UpToDate have greater proportions of citations for hypertension. It is also interesting to note the fluctuation in citation count for the topics hyperlipidemia and CO poisoning.

**Figure 2:**
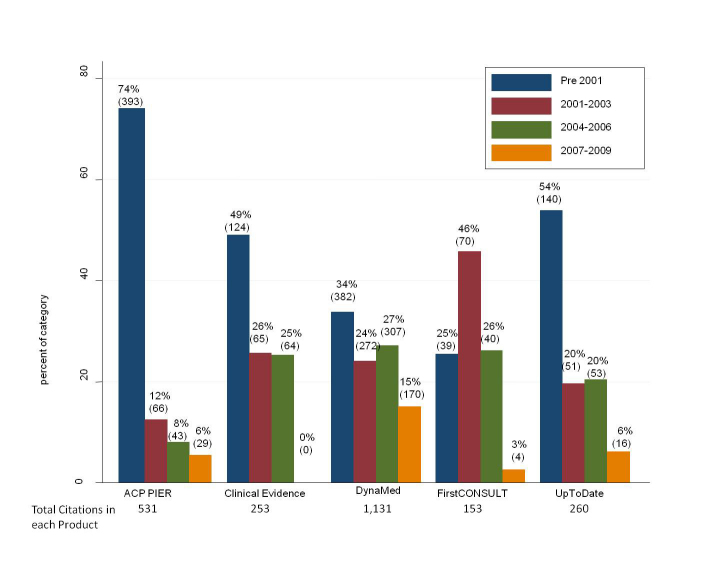
Currency of citations in point-of-care products

The distribution of citations by date for all four products is represented in Figure 2, revealing a general pattern of the pre-2001 group containing the greatest number of citations for each of the products, and the 2007-2009 group containing the fewest. Only FirstCONSULT did not follow this pattern: the 2001-2003 grouping had the greatest number of citations in FirstCONSULT.

Note that two observations are missing from analysis, both Web addresses that are either broken or no longer available, one from Clinical Evidence and one from UpToDate: (1) Clinical Evidence – Office of National Statistics, http://www.statistics.gov.uk, no date, no indication of document to retrieve, from CO Poisoning monograph, (2) UpToDate – www.cdc.gov/nceh/airpollution/carbonmonoxide/cofaq.htm, accessed for UpToDate August 9, 2005, from CO Poisoning monograph.

**Figure 3:**
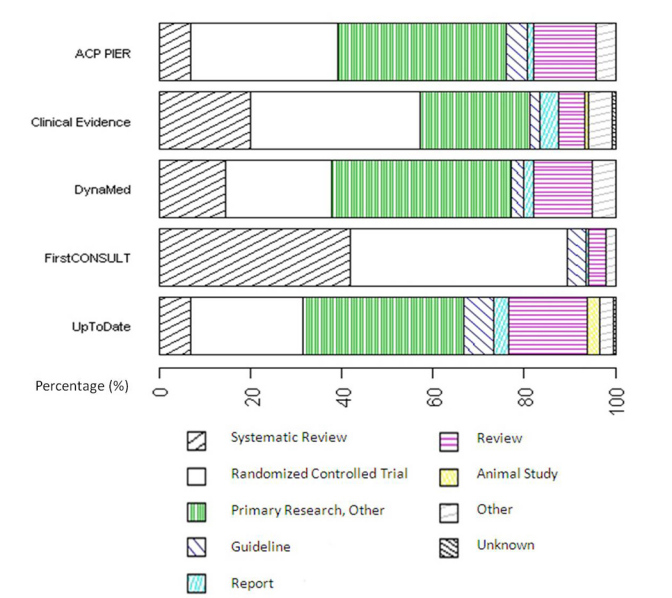
Distribution of publication types found in all topics combined for each point-of-care product (see Table 1 for further details on category definitions)

Figure 3 and Table 3 show the distribution of publication types found in all topics combined for each POC product. FirstCONSULT used the largest proportions of citations with the publication types systematic review and randomized controlled trial. It should be noted that the review publication type category included a small number of evidence reviews, and synopses as defined by Haynes [9], such as Family Physicians Inquiries Network (FPIN) and ACP Journal Club articles. These totaled 7% (20/286) of the total reviews. We found 18 in DynaMed, 1 in FirstCONSULT, and 1 in Clinical Evidence. This was a result of the MEDLINE indexing of these types of articles under the MeSH publication type review.

**Table 3:** Detailed analysis of publication types by point-of-care product (may not total 100% due to rounding)

Publication type	ACP PIER % (n)	Clinical Evidence % (n)	DynaMed % (n)	FirstCONSULT % (n)	UpToDate % (n)	Total % (n)
Systematic review	7.0 (37)	20.1 (51)	14.5 (164)	41.8 (64)	6.9 (18)	14.3 (334)
Randomized controlled trial	32.2 (171)	37.0 (94)	23.3 (263)	47.7 (73)	24.5 (64)	28.5 (665)
Primary research, other	36.9 (196)	24.0 (61)	39.3 (444)	0.0 (0)	35.2 (92)	34.0 (793)
Guideline	4.5 (24)	2.4 (6)	2.9 (33)	3.9 (6)	6.5( 17)	3.7 (86)
Report	1.3 (7)	4.0 (10)	2.0 (23)	0.7 (1)	3.4 (9)	2.1 (50)
Review	13.8 (73)	5.9 (15)	13.0 (147)	4.0 (6)	17.2 (45)	12.3 (286)
Animal study	0.0 (0)	0.8 (2)	0.0 (0)	0.0 (0)	2.7 (7)	0.4 (9)
Other	4.3 (23)	5.1 (13)	5.0 (57)	2.0 (3)	3.1 (8)	4.5 (104)
Unknown	0.0 (0)	0.8 (2)	0.0 (0)	0.0 (0)	0.4 (1)	0.1 (3)
Total	100.0 (531)	100.1 (254)	100.0 (1131)	100.1 (153)	99.9 (261)	99.9 (2330)

An unexpected finding in this study was the very limited overlap between citations across all products, particularly considering the major topics and the summary nature of the information resources.

The monographs for the four topics in all POC products yielded 2330 references. Only two (0.09%) out of the total pool of references were found in all five products. A total of 90.9% (1907/2099) citations were unique, with the topics asthma and hypertension having the greatest number of unique citations across the five POC Products. CO poisoning had the fewest citations overall. The drop-off from the number of unique citations in a POC product to those appearing in additional products is striking: topics appearing in two products fall to the area of 3% and lower (see Table 4).

**Table 4:** Overlap of citations, all topics across all point-of-care products

# Observations^a^	Combined	CO Poisoning	Hyperlipidemia	Hypertension	Asthma
5 times	2 (0.1%)	2 (0.1%)	0 (0.0%)	0 (0.0%)	0 (0.0%)
4 times	4 (0.2%)	1 (0.1%)	2 (0.1%)	0 (0.0%)	1 (0.0%
3 times	25 (1.2%)	3 (0.1%)	8 (0.4%)	8 (0.4%)	5 (0.2%)
2 times	161 (7.7%)	15 (0.7%)	17 (0.8%)	61 (2.9%)	65 (3.1%)
Unique	1907 (90.9%)	147 (7.0%)	269 (12.8%)	670 (31.9%)	830 (39.6%)
Total # citations	2099				

^a^This designation indicates in how many products a reference was found to be cited. For example, 2 times (from the # Observations column) indicates that 161 references of the total 2099 (= 2 + 4 + 25 + 161 + 1907) references (from the Combined column) were found to be cited in two products; 1907/2099 (90.9%) citations were found in only one product. Thus, if the number in the # Observations column is multiplied by the Combined column and added, the final sum is 2330 (= 5 × 2 + 4 × 4 + 3 × 25 + 2 × 161 + 1 × 1907). The overlap of citations was counted by using STATA and Excel.

## Discussion 

This study demonstrates that the characteristics of POC content can be evaluated and used to compare products at a level of detail beyond what is currently available. While time consuming, a bibliographic analysis reveals surprising and critical information about these POC products: they can vary greatly in content, from the raw number of citations, to the types of evidence, to the currency of those citations. 

It was expected that the 2004-2006 grouping of literature would be larger than the 2007-2009 grouping in terms of number of current citations, because it does take some time for systematic reviews and other summarized information resources to be compiled. It was surprising to see that Clinical Evidence contained no citations for the 2007-2009 time period. We can only surmise that this may have been due to the time to update a monograph given the strict editorial policy, and that a separate tab provided access to the latest updated citations that had not yet been incorporated into the monographs.

It is also notable that three of the five POC products show close to 50% or more citations in the pre-2001 range. The number of citations in an entire product database can also have meaning in the interpretation of currency results and in general. Also, many other topics within these products may have greater numbers of current citations. Large sets incorporating older citations may signify access to historic perspectives, while small databases may be closely controlled for other reasons. This was a small test of only four topics and may not be representative of the products as a whole.

The minimal overlap of citations was not only a surprise to us, it was also contrary to expectations expressed by Moed [27]:

A reference list thus contains a certain fraction of unique references, but at the same time there is also a considerable amount of similarity among reference lists.A reference list normally contains a portion of references to documents that are cited in other reference lists as well.

And yet, in a particularly narrow area of medical literature, we found very little overlap.

One important factor this study reveals, as was found in the Abernethy study on compendia [25], is that summary products, such as POC products, vary in content as determined by differences in literature cited for the same topics in different products, quality regarding types of evidence cited, and currency. There are no standards for guidance on developing content for these products. Users should be aware of this and judiciously appraise POC product information content when using resources to obtain information for applying evidence-based practice principles. According to the Haynes 6S pyramid of evidence-based resources, textbook-like summaries, which is how most of the products we evaluated were categorized, fall near the top of the pyramid of evidence-based information resources, which suggests they are among the superior tiers of evidence-based information**.**

However, as reiterated by Strauss and colleagues, authors of *Evidence-Based Medicine: How to Practice and Teach EBM* [40], in a 2009 article entitled “Managing evidence-based knowledge: the need for reliable, relevant and readable resources” [41], not all products that claim to be evidence based are created equal. Strauss and Haynes in this same paper provide guidelines on how to appraise these products and other resources. At the very least, they recommend that the “the minimum criteria for an evidence-based resource would be adherence to the following: Does the resource provide an explicit statement about the type of evidence on which any statements or recommendations are based? Did the authors adhere to these criteria?” [41]. These same questions apply when appraising POC products.

Readers should interpret our findings with some limitations in mind, the most significant being that we analyzed relatively few topics. We did not evaluate the methodological quality of cited studies. Additionally, we collected data within a 6-week time period without regard to the products’ updating schedules. Some assigned publication type classifications may be subject to bias, as the citations were not all independently reviewed by two authors. It should be noted that the proportion of publication types found in each of the POC products may depend on the policies set by their editorial boards. For example, Clinical Evidence follows explicit methods that include evaluating studies from the literature against specific quality criteria prior to inclusion in the monograph.

Sackett et al define EBM as “the conscientious, explicit, and judicious use of current best evidence in making decisions about the care of individual patients” [42]. He further emphasizes that “Evidence-based medicine is not restricted to randomized trials and meta-analyses. It involves tracking down the best external evidence with which to answer our clinical questions” [42]. These critical points about the definition of “evidence-based” should be considered when interpreting our results.

It was our intention to test the potential usefulness of citation/content analysis in this initial study of POC products. Additional tests of more topics and POC products are necessary to confirm and further explore these preliminary results. Further evaluation to look at the quality of citations examined in our study would add strength to the current findings. Furthermore, in light of the minimal citation overlap for topics, it would be helpful to examine the recommendations made for topics with the least overlap, and whether there were differences in recommendations across products for those topics. Finally, it would be beneficial to users if there were standards in product content development for labeling a resource “evidence-based,” as this would minimize variation and arbitrary designations. 

## Multimedia Appendix 1

Topic selection flowchart

## Multimedia Appendix 2

Publication type protocol

## Multimedia Appendix 3

Systematic review classification criteria

## Abbreviations

CO: carbon monoxide
EBM: evidence-based medicine
MeSH: Medical Subject Heading
NLM: National Library of Medicine
POC: point of care
