# Supplementary figures and images for: Type of Evidence Behind Point-of-Care Clinical Information Products: A Bibliometric Analysis
Source: J Med Internet Res. 2011 Feb 18;13(1):e21. doi: 10.2196/jmir.1539 (PMC3221343; doi:10.2196/jmir.1539)

## Appendix 1- Topic Selection Flowchart

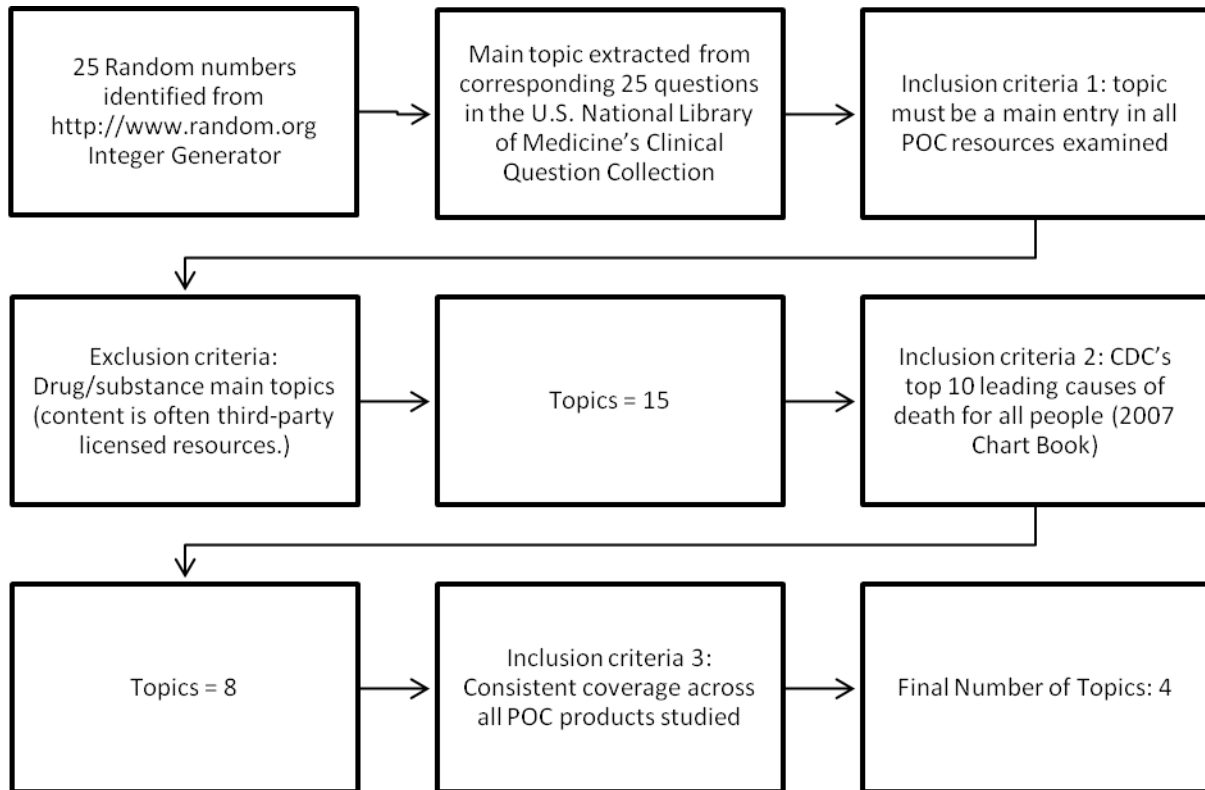

Supplement: Supplementary file 1 [file jmir_v13i1e21_app1.pdf]
