# Supplementary material for: Type of Evidence Behind Point-of-Care Clinical Information Products: A Bibliometric Analysis
Source: J Med Internet Res. 2011 Feb 18;13(1):e21. doi: 10.2196/jmir.1539 (PMC3221343; doi:10.2196/jmir.1539)
Supplement: Supplementary file 2 [file jmir_v13i1e21_app2.pdf]

Appendix 2- Publication Type Protocol

1. First use PubMed MEDLINE to find assigned publication type categories.

2. If not found in PubMed MEDLINE use Embase Emtree terms.

3. If a category placement cannot be decided based on the above, a citation should be checked to determine if it is a study based on: description of a sample being studied (whether it's the number of subjects; or characteristics of the sample), other items in a structured abstract might include objectives, methods, results, discussion, or conclusion.

4. Once an uncategorized citation is determined to be a study based on criteria 3, it can be placed into one of the three main MeSH categories (cohort; cross-sectional; or case-control) based on the MeSH terms for these words

5. If a narrower MeSH term from the MeSH tree hierarchy below is found as a text word in the title, abstract or full text, then the article should be assigned a category for the broader MeSH term. For example, if the word Retrospective is used to describe the study, the broadest category would then be Case-Control Studies in MeSH

    - Epidemiologic Studies
        - Case-Control Studies
            - Retrospective Studies
        - Cohort Studies
            - Follow-Up Studies
            - Longitudinal Studies
            - Prospective Studies
        - Cross-Sectional Studies

6. If not found in Embase.com use agency, publishers, organization's Web site or WorldCat for further investigation of the specific citation in question

7. After all citations are assigned categories, any citation which is indexed as Letter, Comment, or Editorial must have the Title and Abstract examined to determine if the document is reporting on a study based on the following criteria:

    - Description of a sample being studied (whether it's the number of subjects; or characteristics of the sample), other items in the abstract might include objectives, methods, results, discussion, conclusion

8. If the category was modified based on #6, then the original publication type was preserved and documented in the third value in the publication type categorization string (If the original publication type categorization string was

(Other, Letter, x, x), then it would be revised to be publication type (Systematic Review, Systematic Review, Letter, x). The original publication type (Letter) was preserved, but placed in the third value space in the publication type categorization string so that we can keep track that is was originally a Letter publication type.

9. CDC’s Morbidity and Mortality Weekly Reports (MMWR), a government publication, should be classified as Report
