# Supplementary material for: Type of Evidence Behind Point-of-Care Clinical Information Products: A Bibliometric Analysis
Source: J Med Internet Res. 2011 Feb 18;13(1):e21. doi: 10.2196/jmir.1539 (PMC3221343; doi:10.2196/jmir.1539)
Supplement: Supplementary file 3 [file jmir_v13i1e21_app3.pdf]

Appendix 3- Systematic Review Classification Criteria

1. Check the abstract for the criteria listed below.
2. If it is not clear from the abstract if the paper is a systematic review based on criteria, then the full text must be retrieved for evaluation.
3. If it is still not clear, mark with "?" and discuss with 2nd reviewer.
4. If not clear with either reviewer, mark with the original publication type assigned.

Criteria:

- Specific clinical question
- Documentation of methods to identify literature
  - If author states it was "comprehensively" or "systematically" done then that fulfills this criteria
  - If using electronic databases (must use more than one database)
  - If using other resource types, must demonstrate use of multiple sources for identifying studies
- Inclusion and/or exclusion criteria noted
- Synthesis noted
  - Refer to the following references as needed for further details/description on synthesis: Guyatt G, Rennie D, Meade MO, Cook DJ. Users' guides to the medical literature : a manual for evidence-based clinical practice. 2nd ed. New York: McGraw-Hill Medical 2008. Page 525. Table 19.1 or Cook DJ, Mulrow CD, Haynes RB. Systematic reviews: synthesis of best evidence for clinical decisions. Ann Intern Med 1997 Mar 1;126(5):376-380.
